# Supplementary material for: Identification of Gene Biomarkers for Tigilanol Tiglate Content in Fontainea picrosperma
Source: Molecules. 2022 Jun 21;27(13):3980. doi: 10.3390/molecules27133980 (PMC9268252; doi:10.3390/molecules27133980)
Supplement: Supplementary file 1 [file molecules-27-03980-s001.zip › Mitu et al_Supp File 4.pdf]

Supplementary

A

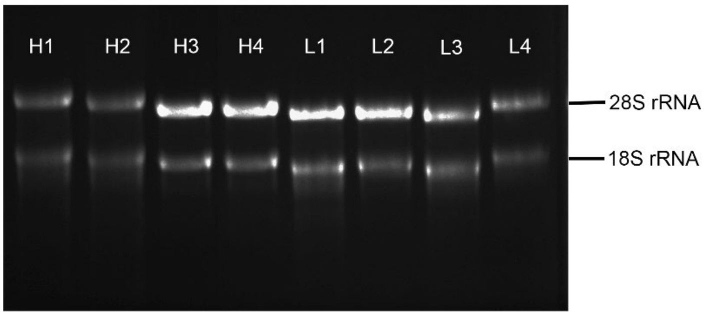

B

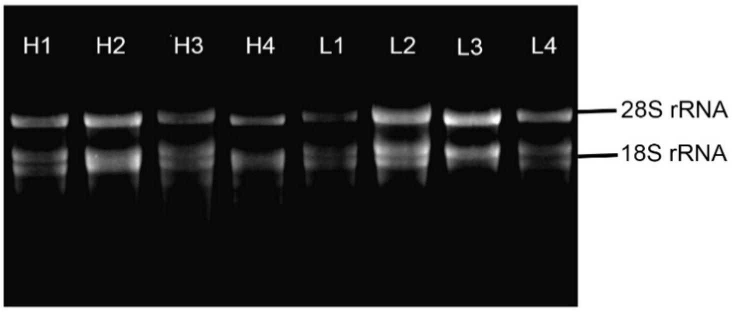

Figure S1. Electrophoretic map of RNA extraction results of each sample; A. Root samples, B. Leaf samples.

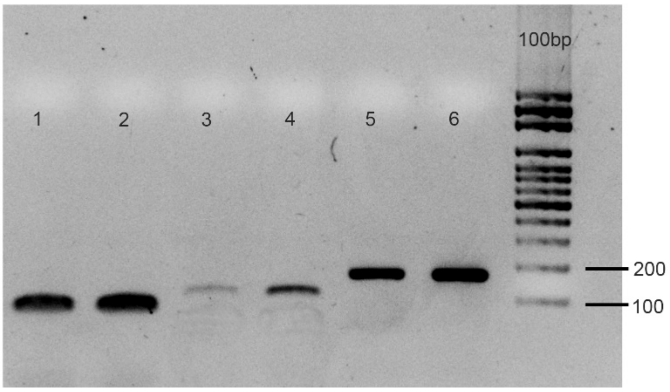

A.

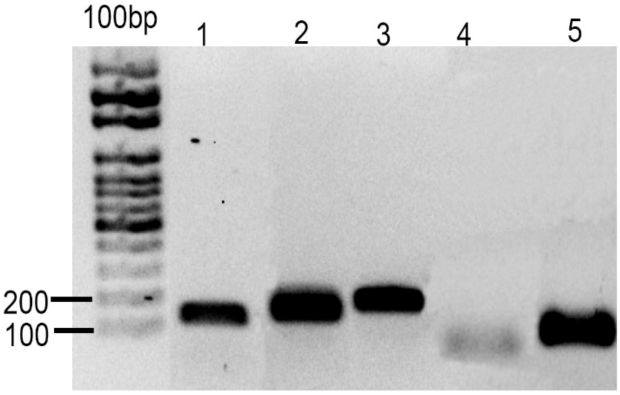

B.

Figure S2. Primer specificity and amplicon size. A. Agarose gel electrophoresis (1.5%) indicates amplification of a single PCR product of the expected size for *PP2A* (1 & 2), *GAPDH* (3 & 4), and *EF1α* (5 & 6) genes in leaf and root samples, respectively. B. Agarose gel electrophoresis (1.5%) indicates amplification of a single PCR product of the expected size for *PSD* (1), *R protein* (2), *ERD* (3), *CYP94C1* (4), and *2-ARD* (5) genes.

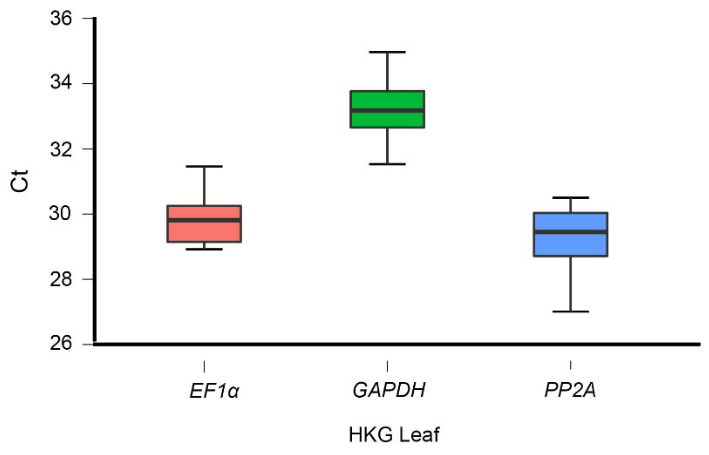

A.

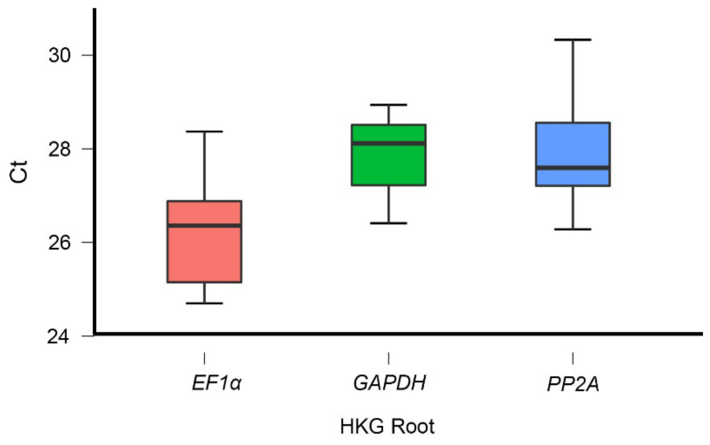

B.

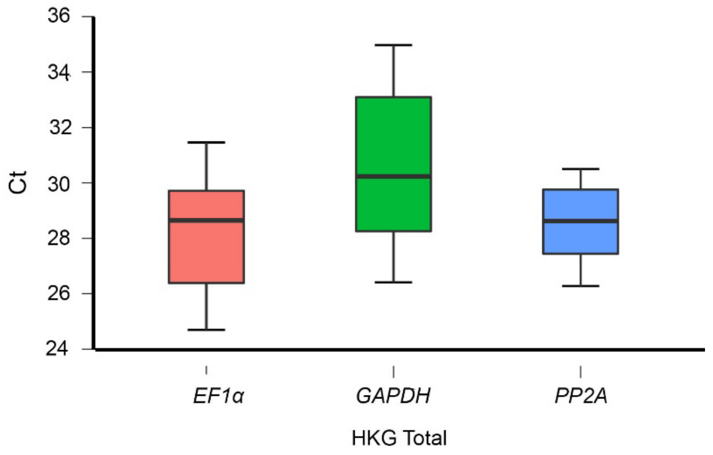

C.

Figure S3. Expression level of the candidate reference genes in leaf (A), root (B), and total (C) samples of Blushwood. Each box plot of Ct value is shown as the first and third quartile and the median value is represented by the horizontal line in the box, whereas whiskers show the maximal and minimal values.

Table S1. Expression stability analysis of reference genes using five algorithms.

Gene	RefFinder		NormFinder		BestKeeper		ΔCt		geNorm	
	**Rank**	**GM**	**Rank**	**SV**	**Rank**	**SD**	**Rank**	**SD**	**Rank**	**MV**
Leaf										
PP2A	1	1.31	1	0.34	3	0.94	1	0.80	1	0.54
GAPDH	2	1.68	2	0.42	2	0.86	2	0.82	1	0.54
EF	3	2.28	3	1.01	1	0.71	3	1.08	3	0.90
Root										
PP2A	1	1.19	1	0.38	2	0.98	1	1.05	1	0.71
GAPDH	3	2.28	3	1.34	1	0.74	3	1.43	3	1.19
EF	2	1.86	2	0.60	3	0.99	2	1.09	1	0.71

Rankings were determined using the parameters GM, geometric mean; SV, stability value; MV, M-Value; and SD, standard deviation.
